# Supplementary material for: Identification of photoperiod-induced specific miRNAs in the adrenal glands of Sunite sheep (Ovis aries)
Source: Front Vet Sci. 2022 Jul 22;9:888207. doi: 10.3389/fvets.2022.888207 (PMC9354845; doi:10.3389/fvets.2022.888207)
Supplement: Supplementary file 1 [file Data_Sheet_1.ZIP › Supplementary file/miRDeep2 file.zip!/miRDeep2 file/Novel_Structure/SP42A2/Novel_173.pdf]

Provisional ID : NW_014639620.1_43990
Score total : 1.7
Score for star read(s) : -1.3
Score for read counts : 0
Score for mfe : 1.4
Score for randfold : 1.6
Score for cons. seed :
Total read count : 81
Mature read count : 81
Loop read count : 0
Star read count : 0

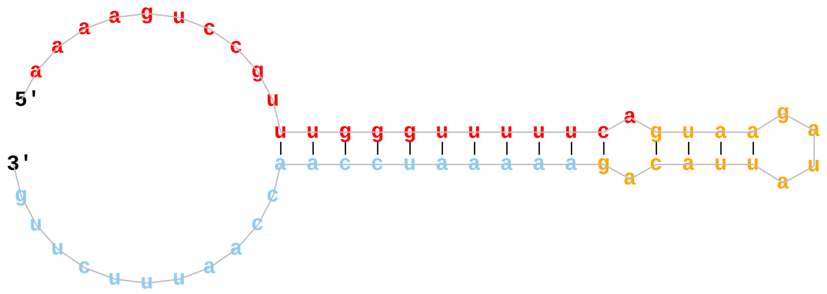

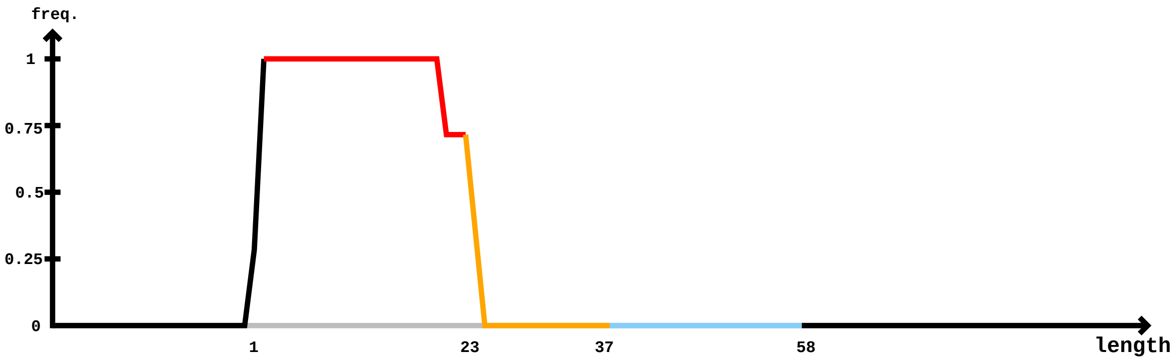

Mature Star

```
5'- uaauauauugguuggcaaaaaaaguccguuuggguuuuucaguaagauauuacagaaaaauccaaccaauuucuuggccaacccaguacuaugauauauuuaauagcuuua   -3'    exp
    .....((((((((((((..((.((((..(((.(((((((((.((((....)))).))))))))))))..)))).)).))))))))))))((((.........)))).....   reads    mm    sample
    ..................aaaaaguUcguuuggguuuu.........................................................................   23       1     seq
    ...................aaaaguccguuuggguuuuuca......................................................................   1        0     seq
    ...................aaaaguUcguuuggguuuuuca......................................................................   57       1     seq
```
